# FPNC Net: A hydrogenation catalyst image recognition algorithm based on deep learning

**DOI:** 10.1371/journal.pone.0300924

**Published:** 2024-05-20

**Authors:** Shichao Hou, Peng Zhao, Peng Cui, Hua Xu, Jinrong Zhang, Jian Liu, Mi An, Xinchen Lin

**Affiliations:** 1 KunLun Digital Technology Co., Ltd, Beijing, China; 2 PetroChina Company Limited, Beijing, China; Newcastle University, UNITED KINGDOM

## Abstract

The identification research of hydrogenation catalyst information has always been one of the most important businesses in the chemical industry. In order to aid researchers in efficiently screening high-performance catalyst carriers and tackle the pressing challenge at hand, it is imperative to find a solution for the intelligent recognition of hydrogenation catalyst images. To address the issue of low recognition accuracy caused by adhesion and stacking of hydrogenation catalysts, An image recognition algorithm of hydrogenation catalyst based on FPNC Net was proposed in this paper. In the present study, Resnet50 backbone network was used to extract the features, and spatially-separable convolution kernel was used to extract the multi-scale features of catalyst fringe. In addition, to effectively segment the adhesive regions of stripes, FPN (Feature Pyramid Network) is added to the backbone network for deep and shallow feature fusion. Introducing an attention module to adaptively adjust weights can effectively highlight the target features of the catalyst. The experimental results showed that the FPNC Net model achieved an accuracy of 94.2% and an AP value improvement of 19.37% compared to the original CenterNet model. The improved model demonstrates a significant enhancement in detection accuracy, indicating a high capability for detecting hydrogenation catalyst targets

## Introduction

With the development and wide application of information technology such as personal computer, Internet and other communication technologies, human society has entered the information age, and the digitalization of information has become the most effective method of information transmission, processing and storage. Digital images have also made great strides in the field of electron microscopy. It is an inevitable trend of modern electron microscope image processing to transform all kinds of imag-es obtained by electron microscope into digital signals for real-time processing and analysis. Transmission electron microscope (TEM) is an important means of material research, which can directly observe the microstructure characteristics of materials. Transmission electron microscopy method can clearly state sulfide phase information of hydrogenation catalyst activity. The statistical results of average ac-cumulation number of active photo crystals and average wafer length have a good correlation with the activity of catalyst. Therefore, whether hydrogenation catalyst electron microscope image segmentation can be accurately identified and key parameters can be determined will directly affect hydrogenation catalyst.

Image segmentation is an important research direction in the field of computer vision. Image segmentation is the semi-automatic or automatic extraction and separation of areas of interest in an image, which lays a foundation for high-level image analysis and understanding, such as model representation of objects of interest, parameter extraction, feature extraction and image recognition [1–4]. Image segmentation is the basis of machine vision, and its accuracy determines the quality of computer vision [5, 6]. As the basis of image analysis, image segmentation is an indispensable step in the process of image processing. Traditional image segmentation methods mainly include: region-based segmentation, edge-based segmentation, threshold-based segmentation and segmentation based on specific theoretical tools, etc. Although it has high computational efficiency and consumes less resources, it is difficult to deal with complex scenes in images [7, 8].

The existing major methods used for image processing to segment electron microscope images include segmentation algorithm based on super pixel [9–12], segmentation method based on wavelet transform and Gaussian difference [13–15], segmentation method based on image block [16–18], and segmentation algorithm based on Otsu [19–21], segmentation algorithm based on neural network [22–25], etc. Otsu algorithm has been widely used as the best algorithm to find the global threshold of image, but it has noise sensitivity and can only segment a single target. Hydrogenation catalyst images often have crystalline impurities and dust, and there are a large number of catalysts with small volumes, and varying lengths and they can be seriously stacked. The above algorithms cannot meet the requirement of segmentation accuracy. Although neural network segmentation algorithm has high accuracy, it requires a large amount of data support, and is faced with issues such as large computation and complex model design [26–31].

On this basis, an image segmentation algorithm of hydrogenation catalyst based on improved morphological trace detection is proposed in this paper. The improved morphology method combined with gradient threshold segmentation method can better retain the catalyst information in the image, improve segmentation accuracy, and provide conditions for the determination of the key parameters of the catalyst in the future. In short, the main contributions of this work are as follows.

1) Proposing a FPNC Net detection algorithm to identify the Catalyst stripe in hydrogenation catalyst images, based on a combination of simple traditional image processing and deep learning methods;
2) Two sets of spatially separable convolution optimized residual modules were used to improve feature extraction capabilities. In addition, FPN is added to fuse shallow and deep features, and CA attention mechanism is introduced to capture cross-channel, direction perception and location sensitive information more effectively to highlight the catalyst target features.
3)The backbone network adds DenseNet to enhance the feature propagation and fuse the features of different scales, and adds an improved ASPP module to adjust the sampling rate and increase the receptive field, so as to further improve the network’s ability to identify and locate targets.

The rest of this paper is organized as follows. The Related Work section describes the image acquisition and image preprocessing. In section Proposed Method, we describe in detail the FPNC Net detection algorithm, the calculation of segmentation result parameters and the statistical analysis methods. Next, The section Hydrogenation catalyst identification algorithm describes the experiments of the algorithm proposed in this paper and gives the experimental results and analysis. Finally, conclusions are drawn in the Conclusions section.

## Related work

### Electron microscope equipment image acquisition

**Fig 1:**
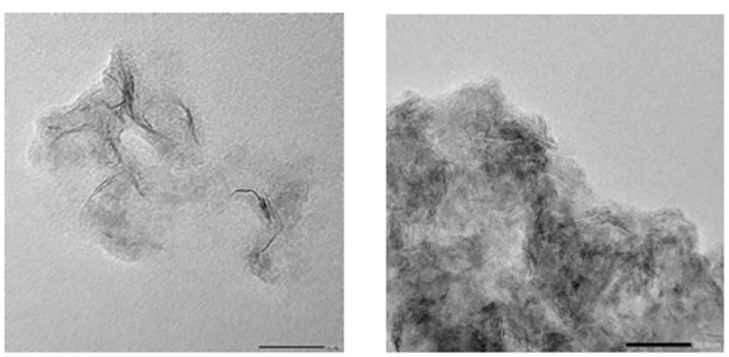
Original image of hydrogenation catalyst.

Hydrogenation catalyst is the catalyst used when the compound and hydrogen addition, the commonly used metal catalyst of Group VIII transition metal elements, metal oxide or sulfide catalyst, complex catalyst, generally for alumina carrier. The electron microscopic image of hydrogenation catalyst magnified 200 times was used as the experimental data. Through the drive module, the device outputs different pulse numbers to complete the collection of microscopic images of the cuddle in the 50cm x 25cm area in the middle of the slide, with a resolution of 1020 pixels x 1020 pixels in jpg format. Part of the original images of the summer cuddle for test are shown in Fig 1.

It can be seen from Fig 1 that the individual hydrogenation catalysts are small and of different lengths. The phase stacking of hydrogenation catalyst is serious. Recognition targets are affected by crystalline impurities and airborne dust. The above image features increase the difficulty of the automatic detection of fungal cradles. After manual selection and preprocessing, a total of 21 420 data sets were obtained. Since the preprocessed image edge would exceed the original image, a background with a color similar to Vaseline was filled in the edge, and the image resolution was modified to 1366.

### Image preprocessing

Sobel is an important processing method in the field of computer vision, which is mainly used to obtain the first-step information of digital images. Its common application and physical significance is edge detection [32]. It is a discrete difference operator used to calculate the gradient approximation of image brightness function. Using this operator at any point in the image will produce the corresponding grayscale vector or its normal vector.

**Fig 2:**
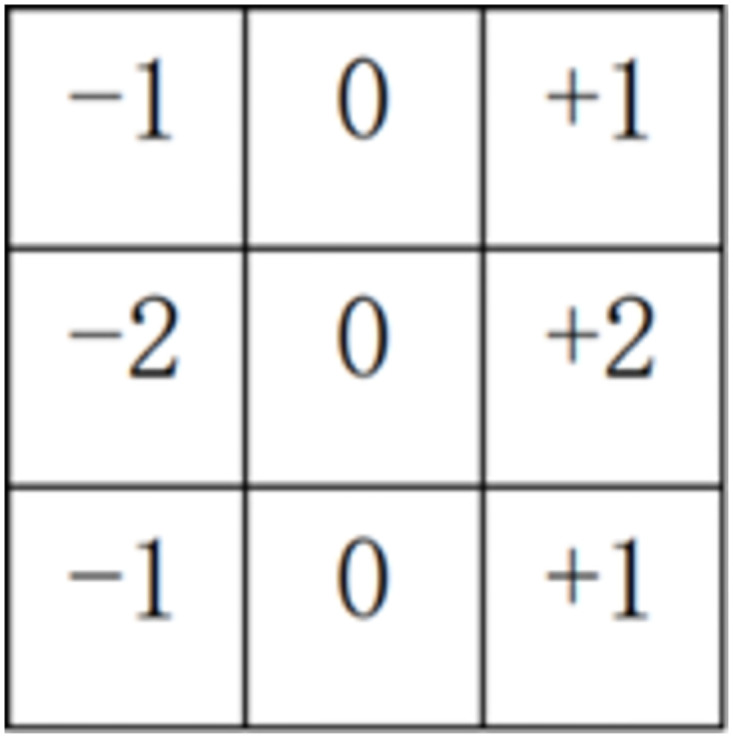
Horizontal template.

**Fig 3:**
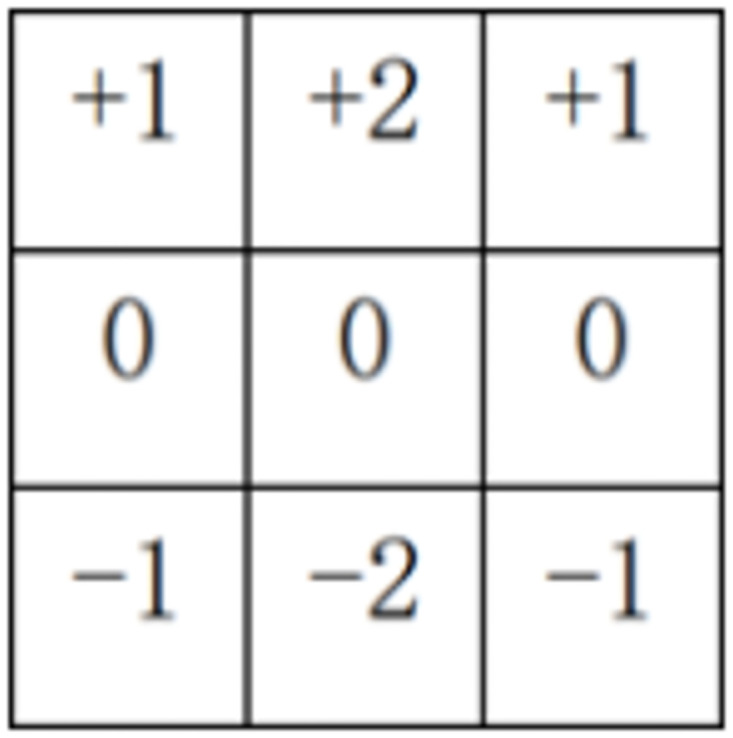
Longitudinal template.

Horizontal and longitudinal gradient templates of Sobel operator are shown in Figs 2 and 3 respectively. The image enhancement part is to improve the quality of the electron microscope image, make it more in line with the visual characteristics of the human eye and facilitate the subsequent operation.

Firstly, histogram equalization is used to make the gray distance of electron microscope image open, make its gray distribution uniform, improve the image contrast. The proportion of each level of gray level in the whole image of pixels was calculated, and the histogram distribution probability of the I-th gray level was denoted as p(i).

Calculate the cumulative value of histogram probability s(i);

$$\begin{array}{c} s(i)=p(0)+p(1)+...+p(i) \end{array}$$ (1)

The pixel gray level of the original image is mapped and transformed. The transformation formula is as follows.

$$\begin{array}{c} ss(i)=int\{[\text{max}(pix)-\text{min}(pix)]\times s(i)+0.5\} \end{array}$$ (2)

Pix refers to the gray value in the original image, ss(i) is the gray level in the equalized image corresponding to the i gray level.

Then the electron microscope image is enhanced based on the fuzzy set theory, that is, the electron microscope image is first mapped from the gray space to the fuzzy domain, the electron microscope image is enhanced by the fuzzy enhancement operator in the fuzzy domain, and then the inverse transformation is carried out to map the fuzzy domain to the gray space, and the fuzzy enhanced image can be obtained [33, 34]. The membership function mapped from gray space to fuzzy domain is as follows.

$$\begin{array}{c} a_{mn}=G(x_{mn})={\left(1+\frac{x_{\text{max}}-x_{mn}}{F_{d}}\right)}^{-F_{e}} \end{array}$$ (3)

In the formula, Fe is the exponential fuzziness factor, with the value of 0.8; Fd is the denominator fuzziness factor, with the value of 128; The fuzzy enhancement operator is as follows.

$$\begin{array}{c} r_{mn}=T^{\left(H\right)}(a_{mn})=T(T^{\left(H-1\right)}(a_{mn})) \end{array}$$ (4)

$$\begin{array}{c} T(a_{mn})=\{\begin{array}{c} 2{\left(a_{mn}\right)}^{2},0\leq a_{mn}\leq u_{c}\\ 1-2{\left(1-a_{mn}\right)}^{2},u_{c}\leq a_{mn}\leq 1 \end{array} \end{array}$$ (5)

In the formula, H represents the number of iteration operations of the function T. Through experiments, it is known that when the fuzzy enhancement operator is used to enhance the electron microscope image, the enhancement effect is the best when the iteration number is 12. uc is the threshold for image enhancement. It can be selected according to the actual situation and is generally 0.5. Then map from fuzzy domain to gray space according to the following inverse transformation formula.

$$\begin{array}{c} y_{mn}=G^{-1}(r_{mn})=x_{\text{max}}-F_{d}[{\left(r_{mn}\right)}^{-\frac{1}{F_{e}}}-1] \end{array}$$ (6)

Finally, the median filter is used to eliminate the isolated noise points. The median filter takes the median value of the pixels covered by the convolution kernel as the pixel value of the anchor point. The 3*3 convolution template is used in this algorithm. That is, the median value of 9 elements in 3 rows and 3 columns is used as the pixel value of the current element. Make the edges of the image sharper. The contrast of the electron microscope image is obviously improved, the noise is effectively suppressed and the details of the electron microscope image are protected.

## Proposed method

### Preparation phase

To improve stripe detection processing time, the features are enhanced by using the traditional algorithm. The visual characteristics of image quality are improved, and complex scenes are filtered to retain only the vertical edges with a single pixel width, which is the information required for the feature extraction stage. The preparation stage is explained in detail as follows.

The basic of mathematical morphology is to keep the main shape information of the image by exploring the structural elements on the target image and removing irrelevant shapes such as noise points and burrs and to obtain the connection between various parts and overall structure of the image [35].

Morphological image processing is performed by moving a structural element in the image and performing a convolution like approach, where the structural element is of arbitrary size and contains a combination of 0 and 1. A specific logical operation is performed between each pixel position and the binary image below it, and the result is output in the image at the position corresponding to that pixel [36]. Corrosion and expansion [37, 38] are the most basic morphological operations in mathematical morphology, and some other morphological operations, such as open and closed operations, are composed of these two basic operations. If f(x,y) is assumed to be the initial image, and g(i,j) to be the structural element, corrosion and expansion can be defined as respectively.

$$\begin{array}{c} f⊖g(i,j)=\text{min}\{f(x-i,y-i)-g(i,j)\} \end{array}$$ (7)

$$\begin{array}{c} f⊕g(i,j)=\text{min}\{f(x-i,y-j)+g(i,j)\} \end{array}$$ (8)

Corrosion is used to eliminate small and meaningless objects, and expansion is used to fill holes in objects. The open and closed operations are established on the basis of the sequential treatment of corrosion and expansion. The open operation is performed through image corrosion followed by image expansion, and the closed operation is performed through image expansion followed by image corrosion. The open and closed operations are defined as follows.

$$\begin{array}{c} f{}^{\circ}g=(f⊖g)⊕g \end{array}$$ (9)

$$\begin{array}{c} f•g=(f⊕g)⊖g \end{array}$$ (10)

The open operation can make the contour of the image smoother, break narrow discontinuities and eliminate fine protrusions. The closed operation can eliminate narrow discontinuity, slender gap and small hole, as well as fill the fracture in the contour line.

### Improved morphology algorithm

#### Feature edge enhancement

Since every curve in the image is a black edge line with strong contrast, the key is to extract the edge with strong contrast. The algorithm proposed in this paper adopts edge detection and morphology algorithm to extract the edge with strong contrast. The algorithm screens the curves that meet the threshold conditions according to the constraints such as shortest pixel, contrast value, length ratio, angle difference and distance, and groups and layers the curves that meet the conditions.

Gabor filter can extract the texture features and edge features of the image well while reducing the influence of lighting and noise on image to a certain extent. The main idea of Gabor filtering method is that different textures generally have different center frequencies and bandwidths, according to which a set of Gabor filters can be designed to filter the texture image. Each Gabor filter allows only the texture corresponding to its frequency to pass smoothly while suppressing the energy of other textures, and then the texture features can be analyzed and extracted from the output results of each filter.

Gabor transform, as an important method for time-frequency analysis, is a convolution of a sinusoidal wave with a Gaussian kernel function in the spatial domain and a Gaussian function after translation in the frequency domain, where the kernel function [39] is defined as:

$$\begin{array}{c} g(x,y;\lambda ,\theta ,\phi ,\sigma ,\gamma )=e^{-\frac{x^{\prime^{2}}+\gamma^{2}y^{\prime^{2}}}{2\sigma^{2}}}\;\text{cos}(2\pi \frac{x^{\prime }}{\lambda }+\phi ) \end{array}$$ (11)

where *x*′ and *y*′ are determined by:

$$\begin{array}{c} x^{\prime }=x\;\text{cos}\;\theta +y\;\text{sin}\;\theta \end{array}$$ (12)

$$\begin{array}{c} y^{\prime }=-x\;\text{sin}\;\theta +y\;\text{cos}\;\theta \end{array}$$ (13)

where x and y represent the pixel coordinate position, λ represents the wavelength of the cosine function, *θ* represents the direction factor of the parallel strip, *σ* represents the standard deviation of the Gaussian function, *γ* represents the aspect ratio of space, and *ϕ* represents the phase deviation of the cosine function.

#### Stripe feature extraction

Refinement is a process of stripping layer by layer, removing some boundary points from the original image, maintaining the original shape until the skeleton of the image is obtained. The input image contour with a certain width is finally changed into one with a width of only one pixel by removing the edge step by step, thus achieving skeleton extraction. The skeleton of the circle is its center, and the skeleton of the lines and isolated points is the circle itself.

First, eight domains centered on the boundary point are found. The center point is denoted as P1, and the 8 points in the domain are denoted as P2, P3..., P9, where P2 is above P1.

1. Mark the boundary points that meet the following conditions:
  (1)2 ≤ *N*(*P*1) ≤ 6
  (2)*S*(*P*1) = 1;
  (3)P2*P4*P6 = 0;
  (4)P4*P6*P8 = 0;
  where N (P1) is the number of non-zero collar points of P1, and S (P1) is P2, P3..., P9 is the num-ber of times the values of these points changing from 0 to 1 in order.
2. Same as the first step, change condition (3) to P2*P4*P8 = 0. Condition (4) is changed to P2*P6*P8 = 0. After all boundary points are checked, all marked points are removed.
3. Step 1 and 2 constitute an iteration until no point meets the marking conditions, and then remaining areas of points constitute a refined skeleton.

**Fig 4:**
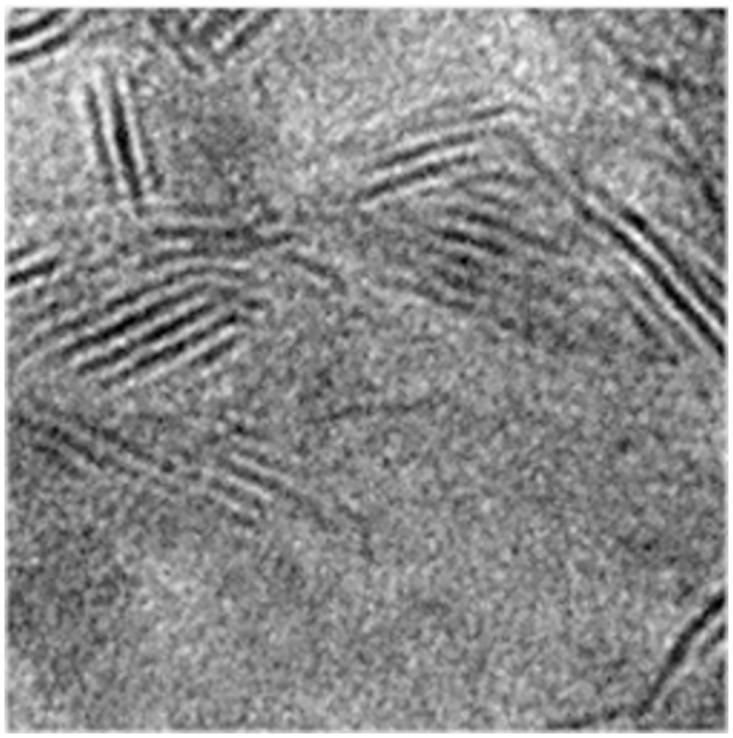
Original image.

**Fig 5:**
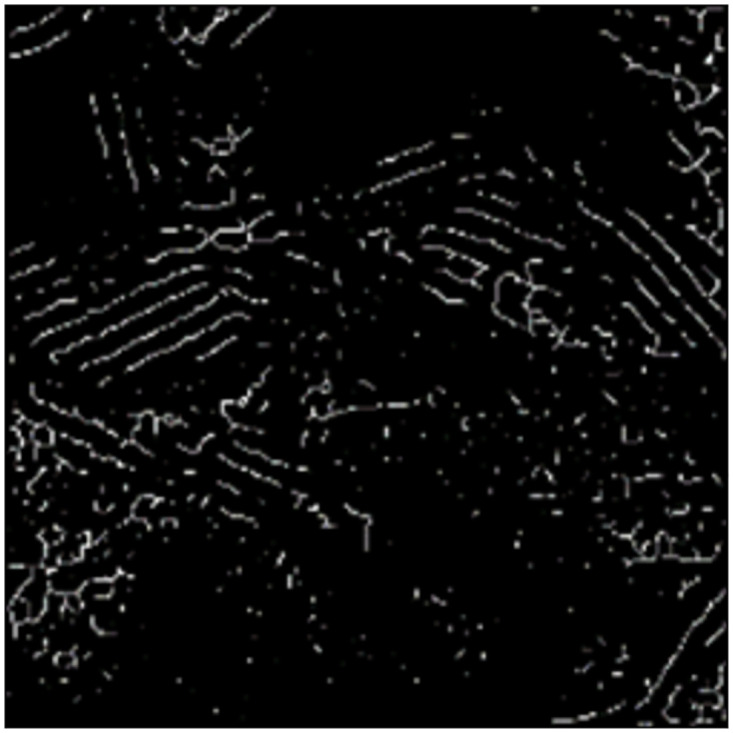
Skeleton line image.

The original electron microscope image and the image after the extraction of skeleton line are shown in Figs 4 and 5.

#### FPNC Net

**Fig 6:**
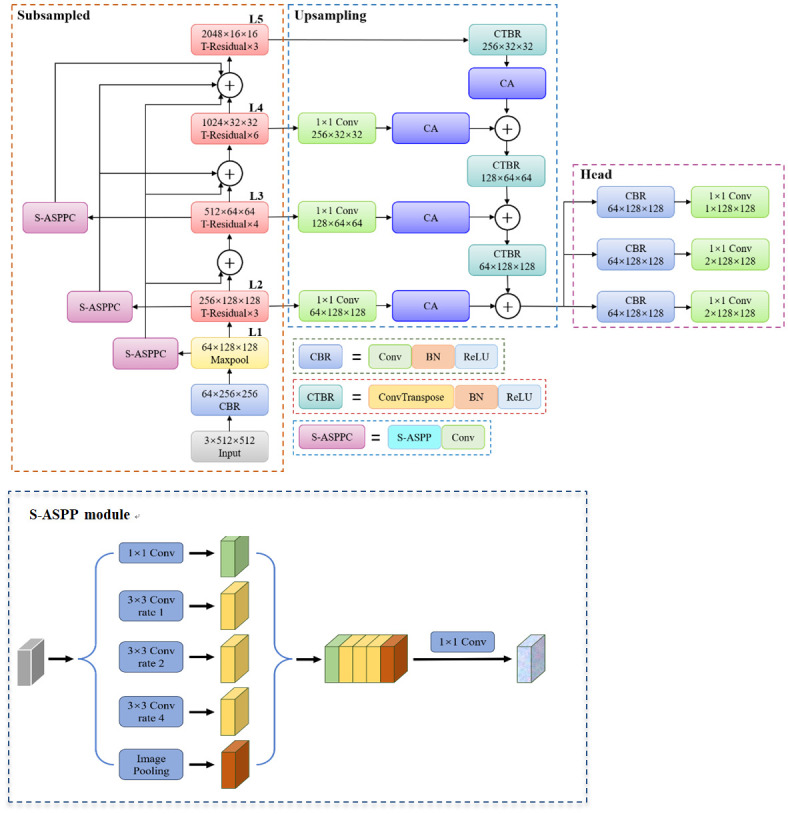
FPNC Net network structure.

The propose method based on the Resnet50 backbone network, the improved CenterNet network is named FPNC Net, and the network structure is shown in Fig 6. In view of the problem of hydrogenation catalyst images with different thickness resulting in different lining, it shows large image background noise, large number of catalysts, small individuals, different lengths and serious stacking phenomenon. First of all, in order to enhance the feature propagation and improve the ability of the network, the feature graph of L1, L2 and L3 first adjusts the sampling rate and increases the receptive field through the S-ASPP module, and then integrates the features of different scales by connecting the convolution of 11 with the input of the back layer. Name the network structure introduced here as DeN. In the upsampling stage, the feature pyramid network uses shallow and deep features to increase the accuracy of the network for small object detection. In addition, the CA attention mechanism is introduced in L2, L3 and L4 to capture cross-channel, and direction perception, and position sensitive information, suppress redundant feature information, improve the efficiency of feature extraction, and enable the model to more accurately locate and identify the objects of interest. Finally, the 33 convolution is used to merge the final feature map to eliminate the overlapping effect brought by the upsampling process, so as to generate the final feature map to complete the detection task.

### Hydrogenation catalyst identification algorithm

#### Catalyst stripe phase length identification rule

The threshold segmentation method combined with maximum connected element is used to extract cuboid black block in the lower right corner of electron microscope image to obtain the pixel length in the corresponding x direction. The actual length of the cell pixel is obtained by dividing the actual length by 20 nm. Then the Euclidean distance of n continuous coordinates on the curve is calcu-lated point by point, and summed up to be the length of the curve.

The shortest pixel and contrast value of the test curve to be tested is defined as Lengthmin and ValueC. A total of m curves that meet the conditions are selected according to the thresholds of Lengthmin and ValueC. The larger the value of Lengthmin, the shorter the curves will be eliminated. The value of ValueC ranges from 0 to 1. The larger the value, the more obscure the curves will be culled.

The formula for calculating the shortest pixel and contrast values is shown as:

$$\begin{array}{c} Length_{\text{min}}=\text{min}(sqrt({\left(x1-x2\right)}^{2}+{\left(y1-y2\right)}^{2})) \end{array}$$ (14)

The coordinates of the two endpoints of the curve are A (x1, y1), B (x2, y2):

Valuec=∑δδi,j2Pδi,j (15)

where *δ*(*i*, *j*) = |*i* − *j*| as the difference in gray scale between adjacent pixels; *P*_*δ*_(*i*, *j*) is the pixel distribution probability, and *δ* is gray difference between adjacent pixels.

#### Catalyst stripe phase identification process

**Fig 7:**
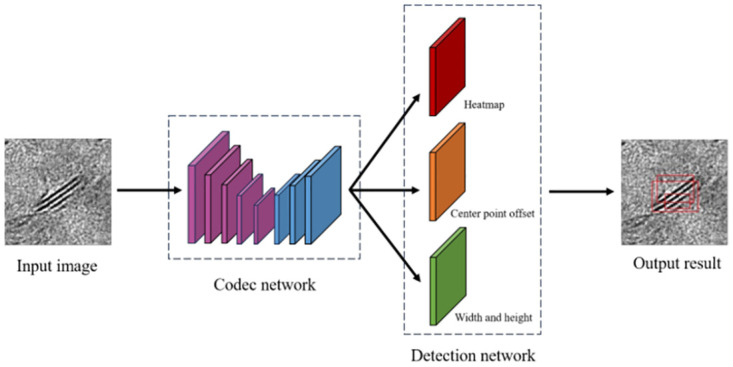
Striker identification model of the hydrogenation catalyst.

The stripe phase recognition model of hydrogenation catalyst is shown in the following Fig 7:

If two curves are similar in length, angle and distance, the two curves are regarded as a group. For m curves to be processed, they can be grouped according to this rule. All similar curves are regarded as a group, and the number of curves in the group is the number of layers.

The length of the two curves is defined as l1 and l2, the center point as c1 and c2, and their length ratio, angle difference and distance respectively as lengththr, anglethr and disthr. Therefore, in order to determine whether the two curves are a group, it is necessary to consider length ratio, angle difference and distance:

The length ratio of the two curves is defined as

$$\begin{array}{c} length_{thr}=\text{max}(l1,l2)/\text{min}(l1,l2) \end{array}$$ (16)

Max () and min () are the maximum and minimum functions respectively. The value ranges from 1 to inf. 1 indicates that curves have the same length. The larger the value, the greater the difference in curve lengths. The threshold of length ratio ranges from 1 to 1.5.

Anglethr is the angle between the starting point and the ending point of two curves, whose value ranges from 0 to 90. 0 indicates the same direction. The larger the value, the greater the difference in direction between the two curves.

The distance between the two curves is defined as

$$\begin{array}{c} dis_{thr}=\text{max}(D(c1,l2),D(c2,l1)) \end{array}$$ (17)

D() is the shortest distance from the center of the first curve to the second curve, whose value represents the maximum value of the shortest distance from the center of one curve to another curve. The value ranges from 0 to inf. 0 means that the distance is 0, and the larger the value is, the greater the distance is. The distance threshold is 20 pixels.

**Fig 8:**
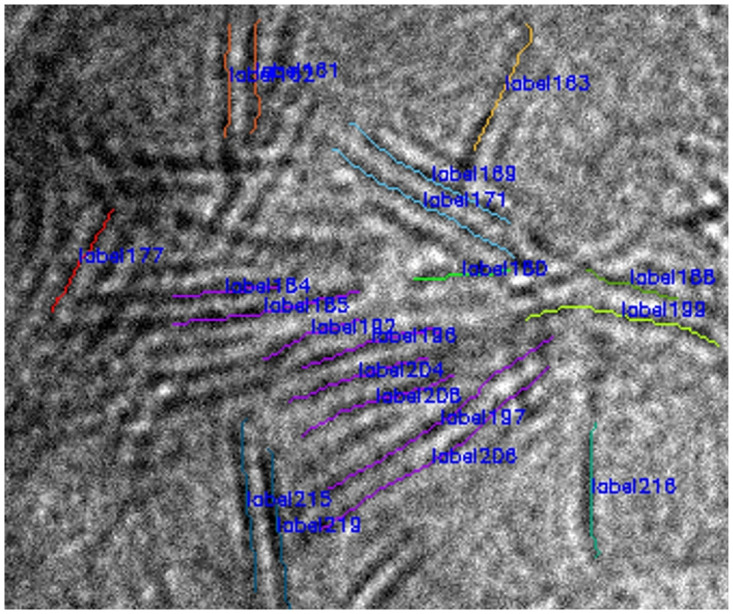
Grouping and layering.

Only when all the above three conditions meet the set threshold can two curves be considered as a group. As shown in Fig 8, the six curves in horizontal direction are the same group.

The electron microscope image acquisition process is often affected by light and noise, and complex background also has a large impact on the segmentation of the catalyst. Therefore, specific algorithm steps in this paper are shown as:

(1) Image enhancement and Gabor filtering are performed on the input hydrogenation catalyst images.
(2) Skeleton line is extracted by OpenCV function.
(3) Curve detection: morphological algorithm is used to process skeleton lines, and each disconnected skeleton line is a curve to be detected.
(4) Sobel operator is used to detect skeleton line endpoints.
(5) According to the endpoint of skeleton line, the whole curve coordinates are extracted in sequence.
(6) The curve length, contrast and other parameters to be detected are calculated.
(7) Skeleton line to be detected is screened.
(8) Grouping and stratification are performed.

## Results

### Experimental dataset

**Fig 9:**
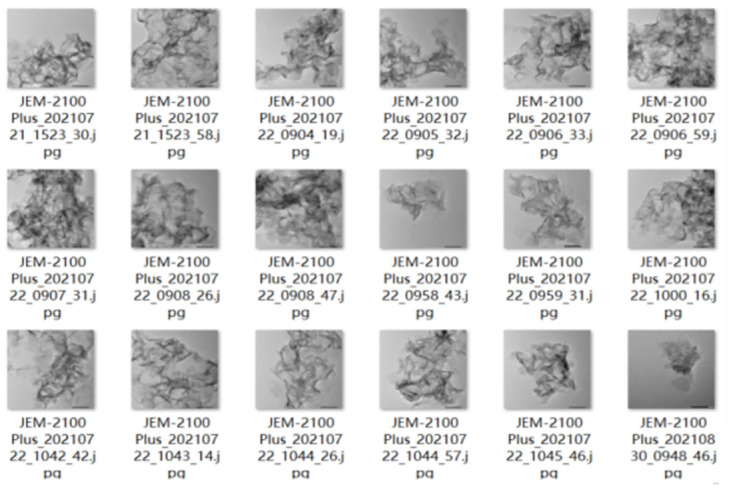
Training set and test set.

6,000 hydrogenation catalyst images were collected with transmission electron microscopy and used as the data set. The data set for network training was randomly divided into training sets and test sets at a 5:1 ratio, as shown in Fig 9.

**Fig 10:**
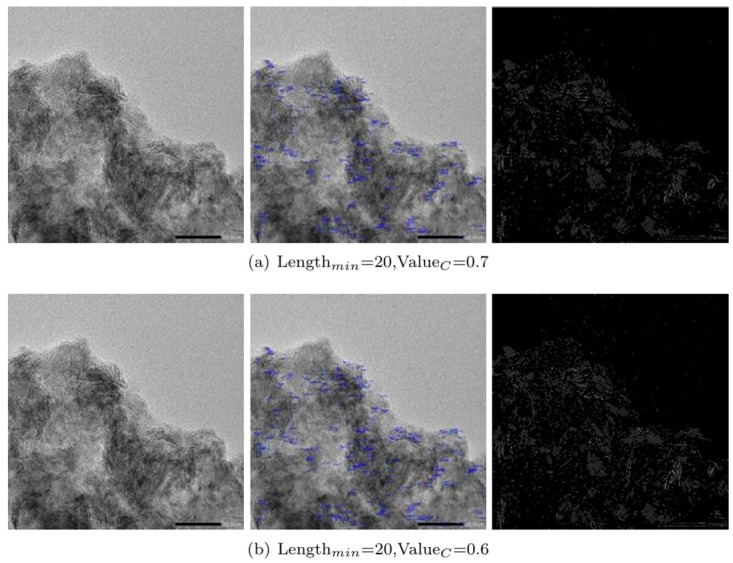
Comparison of experimental results with different parameters (a) to (b).

In order to compare the recognition accuracy under different preprocessing methods, the Batch size was set to 64, and image enhancement was adopted. As shown in Fig 10, HE means the use of color histogram for processing; Origin means the original image; and FST means the use of fuzzy set theory image enhancement for processing. Since FST would lose edge details, the final classification accuracy rate would be lower than that of the original image. As HE could make up for this shortcoming while achieving the highest classification accuracy rate, the HE processing results were used in subsequent experiments.

### Evaluation indicators

Firstly, the following definition is given for Tp Tn Fp Fn .Tp is the number of positive samples detected as positive samples, Fp is the number of negative samples detected as positive samples, and Fn is the number of positive samples not detected. To verify the effectiveness of the algorithm proposed in this study, the hydrogenation catalyst images are tested and evaluated by Precision, Recall, Missed Detection Rate (MDR), False Detection Rate (FDR), and F1 values. The calculation equations are shown below, respectively.

$$\begin{array}{c} Precision=\frac{T_{P}}{T_{P}+F_{P}} \end{array}$$ (18)

$$\begin{array}{c} \text{Re}call=\frac{T_{p}}{T_{P}+F_{N}} \end{array}$$ (19)

$$\begin{array}{c} MDR=\frac{F_{N}}{T_{P}+F_{N}} \end{array}$$ (20)

$$\begin{array}{c} FDR=\frac{F_{P}}{T_{P}+F_{P}} \end{array}$$ (21)

$$\begin{array}{c} F1=\frac{2\text{Re}call\times Precision}{\text{Re}call+Precision} \end{array}$$ (22)

### Experimental results and analysis

#### Experimental parameters

In this paper, the electron microscopic image of hydrogenation catalyst with 200 times magnification is taken by JE0L2100PLUS transmission electron microscope.

Five parameters to be included in the experiment are defined, wherein Lengthmin and ValueC will greatly affect the test result.

(1) Lengthmin is the shortest pixel length of the curve to be detected. The larger the value is, the more short curves will be eliminated in the detection;
(2) ValueC is the contrast value of the curve to be detected. The value ranges from 0 to 1. The larger the value is, the more obscure curves will be eliminated.
(3) lengththr is the length ratio threshold between two curves to be fused, whose value ranges from 1 to 1.5;
(4) disthr is the distance threshold between two curves to be fused, whose value represents the maximum value of the shortest distance between the center of one curve and another curve. 0 means that the distance is 0. The larger the value, the greater the distance. The distance threshold is 20 pixels.
(5) anglethr is the angle between the starting point and the ending point of two curves to be fused. The value ranges from 0 to 90. 0 indicates the same direction. The larger the value, the greater the difference in direction between the two curves, and the angle threshold is 20°.

#### Analysis of experimental results

**Fig 11:**
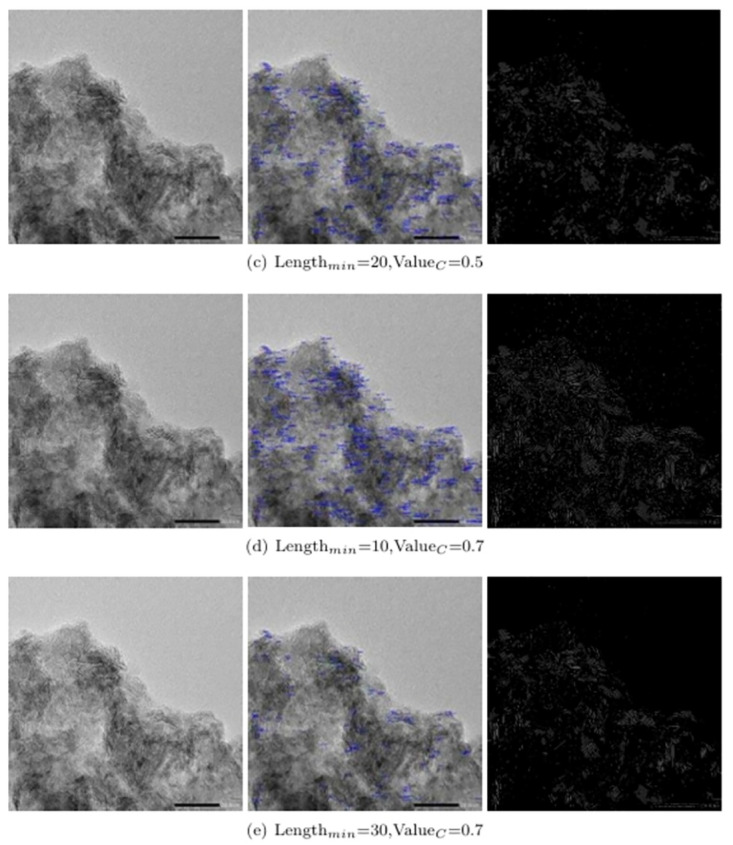
Comparison of experimental results with different parameters (c) to (e).

The comparison of the experimental results of different parameters is shown in Figs 10(a), 10(b) and 11(c) to 11(e), the relationship between Lengthmin, ValueC and each evaluation index is shown in Table 1 and Fig 11, and the detection results are shown in Fig 10.

**Table 1:** Comparison of the results of different parameters evaluation indexes.

Length_*min*_	Value_*c*_	Recall	Precision	MDR	FDR	F1
20	0.5	0.99	0.71	0.01	0.29	0.82
20	0.6	0.98	0.74	0.02	0.26	0.84
20	0.7	0.96	0.76	0.04	0.24	0.84
20	0.8	0.94	0.78	0.06	0.22	0.84
20	0.9	0.92	0.79	0.08	0.21	0.84
24	0.5	0.98	0.74	0.02	0.26	0.84
24	0.6	0.96	0.77	0.04	0.23	0.85
24	0.7	0.93	0.79	0.07	0.21	0.85
24	0.8	0.90	0.81	0.10	0.19	0.84
24	0.9	0.86	0.82	0.14	0.18	0.83
28	0.5	0.95	0.77	0.05	0.23	0.84
28	0.6	0.92	0.79	0.08	0.21	0.84
28	0.7	0.89	0.81	0.11	0.19	0.84
28	0.8	0.85	0.83	0.15	0.17	0.84
28	0.9	0.80	0.85	0.20	0.15	0.82
32	0.5	0.92	0.79	0.08	0.21	0.84
32	0.6	0.88	0.81	0.12	0.19	0.84
32	0.7	0.83	0.83	0.17	0.17	0.83
32	0.8	0.78	0.85	0.21	0.15	0.81
32	0.9	0.72	0.87	0.28	0.13	0.78
36	0.5	0.88	0.81	0.12	0.19	0.84
36	0.6	0.83	0.83	0.17	0.17	0.82
36	0.7	0.77	0.85	0.23	0.15	0.81
36	0.8	0.72	0.87	0.28	0.13	0.78
36	0.9	0.64	0.88	0.36	0.12	0.73

According to the operation results of different parameters in Figs 10(a) to 10(b) and 11(c) to 11(e), some short curves can be eliminated by increasing the shortest pixel length Length_*min*_ of the curve to be tested. If contrast value of the curve to be detected Value_*c*_ is increased, the curve with weak contrast can be obviously screened out.

**Fig 12:**
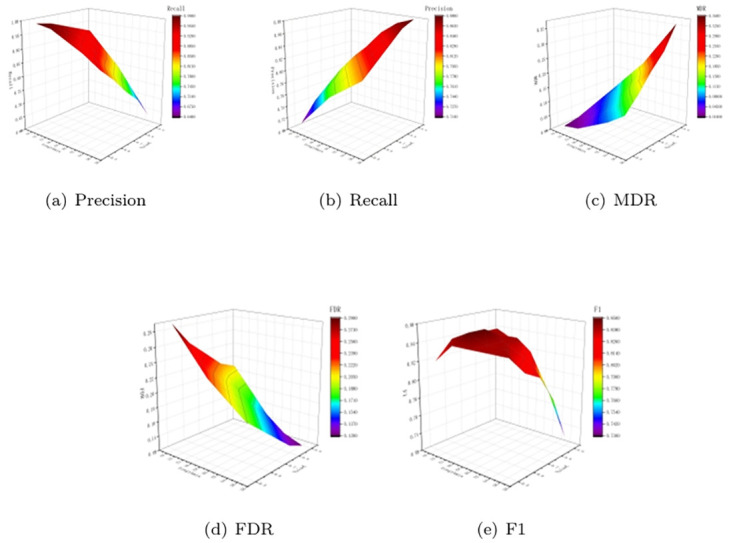
Trend of each evaluation index with Length_*min*_ and Value_*C*_.

As can be seen from Table 1 and Fig 12, increased Length_*min*_ and Value_*c*_ will lead to an increase in the miss detection rate. Although false detection rate will be reduced, accuracy rate will be increased and recall rate will be reduced. Therefore, F1 value is used to evaluate Precision and Recall as a whole, and F1 value of each index is maximum and relatively balanced at Length_*min*_=24 and Value_*c*_=0.7.

**Fig 13:**
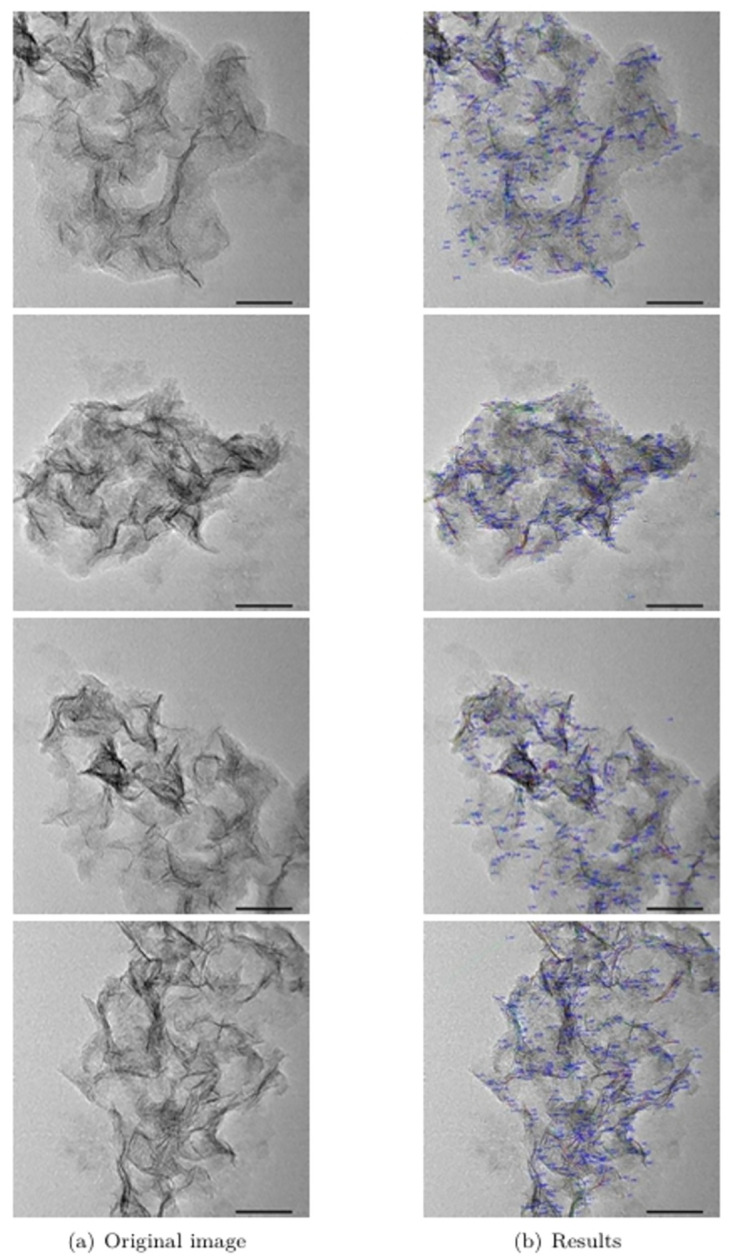
Algorithm results.

The algorithm results for Length_*min*_=24 and Value_*C*_=0.7 are given in Fig 13. The algorithm results and the above evaluation indexes show that the algorithm can effectively segment and detect the hydrogenation catalyst.

CenterNet can locate objects more accurately by predicting the center point of the object, thus improving the detection accuracy. Compared with traditional algorithms and other object detection algorithms (such as YOLO,CornerNet), CenterNet performs better in accuracy. CenterNet adopts an end-to-end model without a complex two-stage correction process, so it can detect objects faster, In addition, it adopts a dense sampling method for center point prediction, reducing the amount of calculation and further improving the detection speed. CenterNet adopts a lightweight network structure, reducing the network parameters, thus reducing the model complexity and computing cost, Due to its simple and efficient characteristics, CenterNet is more easy to deploy and integrate into various systems in practical applications, and has great advantages in practical applications, CenterNet can better deal with the problem that different thicknesses of hydrogenation catalyst images often lead to different levels (crystalline impurities and dust)and the characteristics of numerous catalysts, small individuals, different lengths and serious stacking phenomenon. Parameter selection is shown in Table 2.

**Table 2:** Model training parameters.

Parameter Name	Parameter values
Epoch	200
Batch size	16
Input shape	512x512
Optimizer	Adam
Momentum	0.9
Learning rate decay mode	Cosine annealing
Maximum learning rate	0.0005
Minimum learning rate	5e-06

## Conclusions

To solve the misdetection of hydrogenation catalysts, this paper proposed an improved CenterNet detection model using two different sets of spatially separable convolutions to improve the feature extraction capability. In addition, the feature pyramid network is added to integrate shallow and deep features, and the coordinate attention mechanism is introduced to capture cross-channel, direction perception and location-sensitive information, so as to more accurately locate and identify the objects of interest. Secondly, in the downsampling stage, DenseNet optimization backbone network was used to enhance feature propagation, and the improved ASPP module was added to adjust the sampling rate and increase the receptive field, so as to further improve the ability of the network’s ability to identify and locate targets. The experiments on the test set showed that the AP value of the improved CenterNet model was 20.16,24.80,12.92 percent higher than YOLOv4, SSD, Faster R-CNN detection algorithm in the same experimental environment, and the AP value increased by 19.37 percentage points compared with that of the original CenterNet model. The improved model effectively improves the target detection leakage and misdetection situation, and the detection box is more accurate and compact. Therefore, the improved CenterNet detection model presented here can provide an effective methodological support for hydrogenation catalyst detection. Different models identify the contrast results is shown in Table 3.

**Table 3:** Different models identify the contrast results.

Model	Precision	Recall	MDR	FDR	Number of images
CNN	0.76	0.72	0.37	0.19	1200
FasterR-CNN	0.82	0.79	0.21	0.17	1200
MaskR-CNN	0.87	0.82	0.16	0.25	1200
MSSD	0.81	0.76	0.19	0.12	1200
Centernet	0.88	0.86	0.12	0.07	1200
FPNCNet	0.94	0.89	0.13	0.07	1200
